# Supplementary material for: Functionally coupled ion channels begin co-assembling at the start of their synthesis
Source: eLife. 2026 Jan 15;14:RP106791. doi: 10.7554/eLife.106791 (PMC12807452; doi:10.7554/eLife.106791)
Supplement: Source code 1. [file elife-106791-code1.zip › eLife-VOR-RA-2025-106791/Source code 1.pdf]

This code is available here: https://github.com/jehuang2/SpotScrambler


```
{
 "cells": [
  {
   "cell_type": "code",
   "execution_count": null,
   "id": "ed0e4a8e-6364-4957-bdc0-c0eca4a57133",
   "metadata": {},
   "outputs": [],
   "source": [
    "#to run lines of code between two ''', delete the two '''"
   ]
  },
  {
   "cell_type": "code",
   "execution_count": null,
   "id": "20f8d04a-7871-4e2b-94c4-67cc7dee18c3",
   "metadata": {},
   "outputs": [],
   "source": [
    "#install scikit-image on computer if you have not already\n",
    "'''\n",
    "pip install scikit-image\n",
    "'''"
   ]
  },
  {
   "cell_type": "code",
```

"execution_count": null,
"id": "8b78e095-a09d-4079-b5f9-0cd6a8485c9b",
"metadata": {},
"outputs": [],
"source": [
"import numpy as np\n",
"import random\n",
"from skimage import io, measure, feature\n",
"from PIL import Image, ImageDraw"
]
},
{
"cell_type": "code",
"execution_count": null,
"id": "5076e61b-65dc-49b6-8431-0ae6ef848513",
"metadata": {},
"outputs": [],
"source": [
"#scikit-image has packages tifffile, pillow, networkx, lazy-loader, imageio, and scikit-image\n",
"#scikit-image, aka skimage\n",
"#python pillow= PIL"
]
},
{
"cell_type": "code",
"execution_count": null,
"id": "011c49e7-311c-4adb-8e86-9c8ac596b5b3",
"metadata": {},
"outputs": [],
"source": [

```
    "#Use ImageJ to process your sample image\n",
    "#Outline cell border. Click Analyze> Tools> ROI Manager. Add cell border to ROI Manager. Click More> Save. Save ROI\n",
    "#Click Analyze> Tools> Save XY Coordinates. This will save all the coordinates within the cell border in a .csv file\n",
    "#this will save a .csv spreadsheet of all the coordinates WITHIN the cell border"
   ]
  },
  {
   "cell_type": "code",
   "execution_count": null,
   "id": "38354914-546c-4e0f-9721-824532baa961",
   "metadata": {},
   "outputs": [],
   "source": [
    "def spotscrambler(file, cellmask):\n",
    "    with open(file, \"rb\") as image:\n",
    "        #load binary image from tif file\n",
    "        binary=io.imread(image)\n",
    "       \n",
    "        #label connected regions in image\n",
    "        #note: default is full connectivity (in this case, 2). can change to connectivity =1 or 2\n",
    "        #connectivity 1 is grouping square pixels that share an edge\n",
    "        #connectivity 2 is grouping square pizels that share an edge or corner\n",
    "        labeled=measure.label(binary, connectivity=1)\n",
    "        \n",
    "        #extract properties (area, centroid, coordinates, label, etc) of each labeled region\n",
    "        regions=measure.regionprops(labeled)\n",
    "\n",
    "        #turn regions/spots into circles with the same area, and then get radius in a list\n",
```

```
"       #drawing circles with square pixels means radius is in pixels, which means only integers of
pixels\n",
"       radii=[]\n",
"       for spot in regions:\n",
"           radius=int(np.sqrt(spot.area/np.pi))\n",
"           radii.append(radius)\n",
"       \n",
"       #sort radii list from largest to smallest\n",
"       radii.sort(reverse=True)\n",
"       \n",
"       #print if you want to see all the radii after rounding down to nearest integer\n",
"       '''\n",
"       print(radii))\n",
"       '''\n",
"       \n",
"       #in earlier step, when making radii into integers, some radii less than 1 were turned into zeros\n",
"       #a radius of 1 yields a circle of 5 pixels in area.\n",
"       #smallest possible \"circle\" is made of 1 pixel. therefore smallest possible radius is 0.5.\n",
"       #turn zeros into 0.5 to keep same number of spots\n",
"       for x in range(len(radii)):\n",
"           if radii[x]==0:\n",
"               radii[x]=0.5\n",
"\n",
"       #print to check that radii of zero length were turned into 0.5\n",
"       '''\n",
"       print(radii)\n",
"       '''\n",
"       \n",
"       #print if you want to know how many spots were detected\n",
"       '''\n",
"       print(f\"The length of radii list is {len(radii)}.\")\n",
```

```
"        '''\n",
"        \n",
"    \n",
"    #acquire coordinates within cell perimeter after ImageJ processing. open .csv spreadsheet\n",
"    fillcoords=[]\n",
"    with open(cellmask, \"r\") as mask:\n",
"        csvfile= mask.readlines()[1:]\n",
"        #csvfile is now a list of strings(lines)\n",
"        for string in csvfile:\n",
"            #change strings into list of x and y, excluding third column in csvfile.\n",
"            list=string.split(\",\")[0:2]\n",
"            #x and y are currently strings of numbers. change into float.\n",
"            for i in range(len(list)):\n",
"                list[i]=float(list[i])\n",
"            #append each list of x, y floats to fillcoords list\n",
"            fillcoords.append(list)\n",
"\n",
"    #create a new image with size of desired pixels\n",
"        #binary.shape gives dimensions (pixels) of binary image- height by width\n",
"        image_size=(binary.shape[1], binary.shape[0])\n",
"        #Image.new makes images with dimensions width by height\n",
"        new_image=Image.new('L', image_size, (0))\n",
"\n",
"    #iterate through list of radii, pick random coordinate for circle centers\n",
"    #check if new circle centers will overlap with existing circles\n",
"    new_center_coords=[]\n",
"    #generate center coordinate for first/largest circle\n",
"    tentativecenter=random.choice(fillcoords)\n",
"    new_center_coords.append(tentativecenter)\n",
"    #now generate center coordinates for all the rest of the circles\n",
```

```
    "    for i in range(1,len(radii)):\n",
    "        #tentativecenter needs to exist outside of while loop. \n",
    "        #if tentativecenter only in while loop, tentativecenter doesn't exist once you break out of while loop.\n",
    "        #assuming overlap\n",
    "        overlap=True\n",
    "        while overlap==True:\n",
    "            overlapcount=0\n",
    "            tentativecenter=random.choice(fillcoords)\n",
    "            #checking if new circle center overlaps with any existing circles\n",
    "            for j,[x,y] in enumerate(new_center_coords):\n",
    "                distance= (np.sqrt(((tentativecenter[0] - x)**2) + ((tentativecenter[1] - y)**2)))\n",
    "                shortestdistance= radii[i]+radii[j]\n",
    "                if distance >= shortestdistance:\n",
    "                    continue\n",
    "                if distance < shortestdistance:\n",
    "                    #overlaps\n",
    "                    overlapcount=overlapcount+1\n",
    "                    overlap=True\n",
    "                    #break out of for loop\n",
    "                    break  \n",
    "            if overlapcount==0:\n",
    "                overlap=False\n",
    "                #exits while loop\n",
    "            if overlapcount>0:\n",
    "                overlap=True\n",
    "                #goes back to top of while loop\n",
    "        new_center_coords.append(tentativecenter)\n",
    "    #print if you want to know how many new circles will be generated. should match the number of original spots\n",
    "    '''           \n",
```

```
    "    print(f\"The length of new_center_coords is {len(new_center_coords)}\")\n",
    "    '''\n",
    "\n",
    "    #draw circles at new coordinates\n",
    "    circlecount=0\n",
    "    draw= ImageDraw.Draw(new_image)\n",
    "    for i, [x,y] in enumerate(new_center_coords):\n",
    "        if radii[i] != 0:\n",
    "            draw.circle([x,y],radii[i], fill=255)\n",
    "            circlecount=circlecount+1\n",
    "            \n",
    "    #print if you want to know how many circles were drawn. should match number of center coordinates\n",
    "    '''\n",
    "    print(circlecount)\n",
    "    '''\n",
    "\n",
    "    #save new image as tif file\n",
    "    new_image.save(f\"{file}.SCRAMBLE.tif\")"
   ]
  },
  {
   "cell_type": "code",
   "execution_count": null,
   "id": "f248f754-2ecb-4cc6-b06b-2d3bfa0fa796",
   "metadata": {},
   "outputs": [],
   "source": [
    "import os\n",
    "imagedirectory= \"pathtoimagefolder\"\n",
    "csvdirectory= \"pathtocsvfolder\""
```

```
   ]
  },
  {
   "cell_type": "code",
   "execution_count": null,
   "id": "a0bcfb0f-334c-4b6a-aeb1-91f21984d8a1",
   "metadata": {},
   "outputs": [],
   "source": [
    "#images and csv files can be in different folders or same folder. doesn't matter.\n",
    "imagelist=[]\n",
    "csvlist=[]\n",
    "for name in os.listdir(imagedirectory):\n",
    "    if name.endswith(\".tif\"):\n",
    "        imagelist.append(f\"{imagedirectory}/{name}\")\n",
    "for name in os.listdir(csvdirectory):\n",
    "    if name.endswith(\".csv\"):\n",
    "        csvlist.append(f\"{csvdirectory}/{name}\")\n",
    "#sort your imagelist and csvlist so that they pair correctly\n",
    "imagelist.sort()\n",
    "csvlist.sort()\n",
    "\n",
    "#print to check that all your files/order are correct\n",
    "\"\"\"\n",
    "print(imagelist)\n",
    "print(csvlist)\n",
    "\"\"\"\n",
    "\n",
    "#print to check that length of both lists are the same\n",
    "\"\"\"\n",
```

```
    "print(len(imagelist))\n",
    "print(len(csvlist))\n",
    """"
   ]
  },
  {
   "cell_type": "code",
   "execution_count": null,
   "id": "9eafbd64-18e9-4ef0-81d6-487c559d113b",
   "metadata": {},
   "outputs": [],
   "source": [
    "#to use SpotScrambler on whole folders\n",
    "for i in range(len(imagelist)):\n",
    "    spotscrambler(imagelist[i], csvlist[i])"
   ]
  },
  {
   "cell_type": "code",
   "execution_count": null,
   "id": "a3b99e51-8e82-47f8-9ac9-431d504d66a0",
   "metadata": {},
   "outputs": [],
   "source": [
    "#to use SpotScrambler one image at a time\n",
    "#function takes two inputs. first input is binary image of spots. second input is .csv file of cell surface
area\n",
    "\n",
    "spotscrambler(\"pathtobinaryimage\",\"pathtocsvfile\")"
   ]
  }
```

],
"metadata": {
"kernelspec": {
"display_name": "Python 3 (ipykernel)",
"language": "python",
"name": "python3"
},
"language_info": {
"codemirror_mode": {
"name": "ipython",
"version": 3
},
"file_extension": ".py",
"mimetype": "text/x-python",
"name": "python",
"nbconvert_exporter": "python",
"pygments_lexer": "ipython3",
"version": "3.11.5"
}
},
"nbformat": 4,
"nbformat_minor": 5
}
